# Pancreatic enzymes digest obstructive meconium from cystic fibrosis pig intestines

**DOI:** 10.3389/fped.2024.1387171

**Published:** 2024-04-11

**Authors:** Gopinathan Gangadharan Nambiar, Sussette Gonzalez Szachowicz, Christian F. Zirbes, Jared J. Hill, Linda S. Powers, David K. Meyerholz, Ian M. Thornell, David A. Stoltz, Anthony J. Fischer

**Affiliations:** ^1^ Stead Family Department of Pediatrics, University of Iowa, Iowa City, IA, United States; ^2^ Department of Pediatrics, East Tennessee State University, Johnson City, TN, United States; ^3^ Department of Pediatrics, University of Iowa, Iowa City, IA, United States; ^4^ Department of Internal Medicine, University of Iowa, Iowa City, IA, United States; ^5^ Department of Pathology, University of Iowa, Iowa City, IA, United States

**Keywords:** meconium, cystic fibrosis, meconium ileus, pancreatic enzymes, mucus, mucolytic, reducing agents, contrast enemas

## Abstract

**Introduction:**

Meconium ileus (MI) is a life-threatening obstruction of the intestines affecting ∼15% of newborns with cystic fibrosis (CF). Current medical treatments for MI often fail, requiring surgical intervention. MI typically occurs in newborns with pancreatic insufficiency from CF. Meconium contains mucin glycoprotein, a potential substrate for pancreatic enzymes or mucolytics. Our study aim was to determine whether pancreatic enzymes in combination with mucolytic treatments dissolve obstructive meconium using the CF pig model.

**Methods:**

We collected meconium from CF pigs at birth and submerged it in solutions with and without pancreatic enzymes, including normal saline, 7% hypertonic saline, and the reducing agents N-acetylcysteine (NAC) and dithiothreitol (DTT). We digested meconium at 37 °C with agitation, and measured meconium pigment release by spectrophotometry and residual meconium solids by filtration.

**Results and discussion:**

In CF pigs, meconium appeared as a solid pigmented mass obstructing the ileum. Meconium microscopically contained mucus glycoprotein, cellular debris, and bile pigments. Meconium fragments released pigments with maximal absorption at 405 nm after submersion in saline over approximately 8 h. Pancreatic enzymes significantly increased pigment release and decreased residual meconium solids. DTT did not improve meconium digestion and the acidic reducing agent NAC worsened digestion. Pancreatic enzymes digested CF meconium best at neutral pH in isotonic saline. We conclude that pancreatic enzymes digest obstructive meconium from CF pigs, while hydrating or reducing agents alone were less effective. This work suggests a potential role for pancreatic enzymes in relieving obstruction due to MI in newborns with CF.

## Introduction

Newborn babies with cystic fibrosis (CF) are at risk of developing meconium ileus (MI), a life-threatening obstruction of the intestines that occurs in approximately 15%–20% of newborns with CF (1–3). Most newborns with CF who suffer from MI have *CFTR* mutations with relatively low residual CFTR function, and the vast majority are pancreatic insufficient. In CF pigs, MI can be prevented by expressing CFTR in intestinal cells (4). Similarly, CFTR modulator drug treatment *in utero*, which would increase CFTR function in both the intestines and pancreas, protects against MI in the ferret model of CF (5). However, except for rare cases (6), using CFTR modulators to prevent MI in newborn babies with CF remains problematic. Many cases of MI from CF are unexpected, owing to a lack of prenatal genetic diagnosis. While some fetal ultrasound findings may suggest MI, the sensitivity and specificity of this method is inadequate to predict MI (1, 2). Thus, newborns with CF can present with symptoms of abdominal distension, bilious vomiting, and shock, which indicate life-threatening MI. Furthermore, many newborns with MI may be ineligible for CFTR modulator therapy due to their genotype.

To relieve MI, many centers use therapeutic enemas which typically include radiographic contrast media to visualize the extent of obstruction and reduce the obstruction. Some protocols include hyperosmolar solutions or reducing agents such as N-acetylcysteine (NAC) to disrupt chemical bonds in meconium. While some have reported successful medical treatment of MI with enemas, the treatment solutions used have not been studied in randomized controlled trials. As most cases of MI ultimately require surgery (2, 7), it is likely that current medical treatments for MI are ineffective.

Surgery is the definitive intervention for MI, but it has associated risks. Newborns with MI can suffer immediate or delayed postoperative complications (1, 8). Babies with MI have prolonged hospitalization in the neonatal intensive care unit (9), and some develop short bowel syndrome from the loss of extensive segments of small intestine (9). These children will ultimately depend upon parenteral nutrition through a central line (10). Short bowel syndrome combined with pancreatic insufficiency contribute to growth failure in children with CF, and infants requiring parenteral nutrition can suffer from central line-associated bloodstream infections (11). Other late surgical complications include intestinal obstruction due to post-surgical adhesions (12, 13). Thus, there is an unmet need to improve medical treatments for MI. Ideally, these treatments would be genotype-agnostic and reduce the risk of long-term surgical complications.

The goal of this study was to determine whether common treatments for MI are effective in breaking down obstructive meconium. We studied newborn pigs with complete loss of CFTR by homozygous deletion of exon 10 (referred to as *CFTR*^−/−^) or severe loss of CFTR function by F508del knock-in mutations (*CFTR*^Δ*F508/*Δ*F508*^) that consistently develop MI with a severe intestinal obstruction that would require surgery for survival (14, 15). We hypothesized that reagents that disrupt the chemical bonds of meconium would disintegrate it over time. Further, as most babies with CF and CF pigs are pancreatic insufficient, the pancreatic enzymes that may normally digest meconium would be deficient. Therefore, we hypothesized that exogenous pancreatic enzyme supplementation could improve digestion of obstructive meconium from CF pigs.

## Methods

### Animals

Animal care and procedures were approved by the Animal Care and Use Committees at the University of Iowa (IACUC approved protocol number # 0081121). We obtained male and female *CFTR*^−/−^ and *CFTR*^ΔF508/ΔF508^ piglets from Exemplar Genetics. Individual pigs used in this study are listed in Supplementary Table S1. We euthanized piglets with pentobarbital sodium-phenytoin sodium (Euthasol, Virbac, Fort Worth, TX). At necropsy, we examined the gut to identify the site of obstruction. We excluded piglets that had evidence of intestinal perforation or peritonitis. We removed the intestines from the abdominal cavity and opened the intestines at the site of obstruction to collect meconium from the dilated segment proximal (orad) of the obstruction. We stored meconium at 4 °C in isotonic saline until the time of experimentation.

### Histopathology

Archived formalin-fixed and paraffin embedded CF pig intestinal tissues (*n *= 5 newborn CF pigs) were identified, and proximal and distal segments of MI obstructions were morphologically examined. Additionally, we preserved proximal segments of MI obstruction (*n *= 3 CF pigs) in 10% neutral buffered formalin for ∼5–7 days. Fixed intestinal segments were paraffin embedded and stained with diastase pretreated Periodic Acid Schiff (dPAS) to visualize glycoproteins vs. appropriate controls (16).

### Chemical reagents

Normal saline was obtained from Baxter. Powdered pancreatic enzymes (Epizyme Product # NDC 068720-023-12) were obtained from VET Brands International. Each teaspoon (2.8 g) of powdered Epizyme preparation contains lipase (71,400 USP Units), protease (388,000 USP Units) and amylase (460,000 USP Units). Other chemical reagents were obtained from Fisher Scientific.

### Meconium digestion

We used a razor to cut CF meconium into small pieces (∼5–10 mm) and weighed them. The typical weight for these pieces was 3–5 g. We placed the weighed meconium pieces in vented 14 ml Falcon tubes and submerged the meconium in 3 ml of test solutions, including normal saline, 7% NaCl, 1 M NAC (as free acid) in water, or 1 M DTT in water. We assumed there would be a stoichiometric relationship between NAC or DTT with the protein disulfide bonds in the meconium. Therefore, we selected high concentrations of NAC and DTT so that these reducing agents would not be limiting reagents. To test the effect of pancreatic enzymes, we added 10 mg/ml of Epizyme powdered pancreatic enzymes to some of the test solutions. We incubated the submerged CF meconium at 37 °C for 16 h. Because mechanical forces could contribute to digestion in normal physiology, we agitated the solutions in an orbital shaker at 250 RPM (New Brunswick Scientific Excella E25).

### Meconium pigment release

After meconium digestion, we allowed meconium solids to sediment on the benchtop for 30 min. We measured the release of colored pigments from the meconium by sampling 200 *µ*l of the supernatant and transferring it to a clear 96 well plate (Corning). We obtained the absorbance spectrum of the supernatant for each sample using a SpectraMax i3x plate reader (Molecular Devices) recording wavelengths between 300 and 700 nm at 5 nm intervals. We used the corresponding test solutions as blanks for each sample. Because the peak in meconium pigment absorbance was near 405 nm, we used A_405_ to compare pigment release between different conditions. We used 10 pigs to compare test solutions (5 males, 5 females, all *CFTR*^−/−^).

### Residual meconium solids

After sampling the supernatant, we measured undigested residual meconium solids by decanting the meconium and test solution onto pre-weighed Whatman #1 filters (GE Healthcare Life Sciences) placed in a Büchner funnel under vacuum. To remove residual salts and other soluble materials, we rinsed the filters with 50 ml of deionized water. We air-dried the filters and measured the dry weight of residual meconium on an analytical balance. We divided the dry weight of the residual filtered meconium by the original weight of the meconium piece prior to treatment to obtain the percentage of residual weight for each treatment group. We photographed residual meconium on each filter paper. The same 10 pigs were used to compare residual solids after treatment with test solutions.

### pH dependence of meconium digestion

We modified our protocol to determine the optimal pH for dissolving obstructive meconium. We prepared a stock solution of 130 mM NaCl buffered with 10 mM 2-N-morpholinoethanesulfonic acid (MES), 10 mM N-(2-hydroxyethyl)piperazine-N′-2-ethanesulfonic acid (HEPES), and 10 mM tromethamine (Tris). We then divided this stock solution into 5 equal aliquots and added either NaOH or HCl to adjust the solution to the desired pH (4.50, 5.50, 6.50, 7.50, or 8.50). We tested the pH dependence of meconium digestion using 6 additional CF pigs (3 males, 3 females, 5 *CFTR*^−/−^ and 1 *CFTR*^ΔF508/ΔF508^) that were independent of the studies above, using the same methods.

### Pancreatic enzyme dose response for meconium digestion

We tested pancreatic enzyme concentrations ranging from 1 mg/ml to 10 mg/ml mixed using the pH 7.50 buffer described above. We used 6 additional newborn CF pigs (3 male, 3 female, independent of the studies above, 5 *CFTR*^−/−^ and 1 *CFTR*^ΔF508/ΔF508^) for these studies with the same methods.

### Kinetics of meconium digestion

We studied the time-response of enzymatic digestion of CF meconium using 4 mg/ml of pancreatic enzymes in normal saline buffered with the pH 7.50 buffer. We obtained meconium from an additional 6 newborn *CFTR*^−/−^ pigs for this study (5 male, 1 female). Meconium solids were submerged either in pH of 7.50 normal saline and incubated at 37  °C with or without 4 mg/ml of pancreatic enzymes. We sampled the supernatant from each condition at two-hour intervals for 16 h. Each sample was measured vs. the corresponding blank solution.

### Statistical analysis

We used GraphPad Prism version 9 for statistical testing. To examine the effects of treatment solution and pancreatic enzymes, we used 2-way repeated measured ANOVA, matching samples taken from the same animal. For post-hoc analysis, we used Holm-Sidak's multiple comparisons test comparing other treatment solutions vs. 0.9% NaCl and enzyme treated samples vs. no enzyme controls.

## Results

### Morphology of meconium ileus in CF pigs

**Figure 1:**
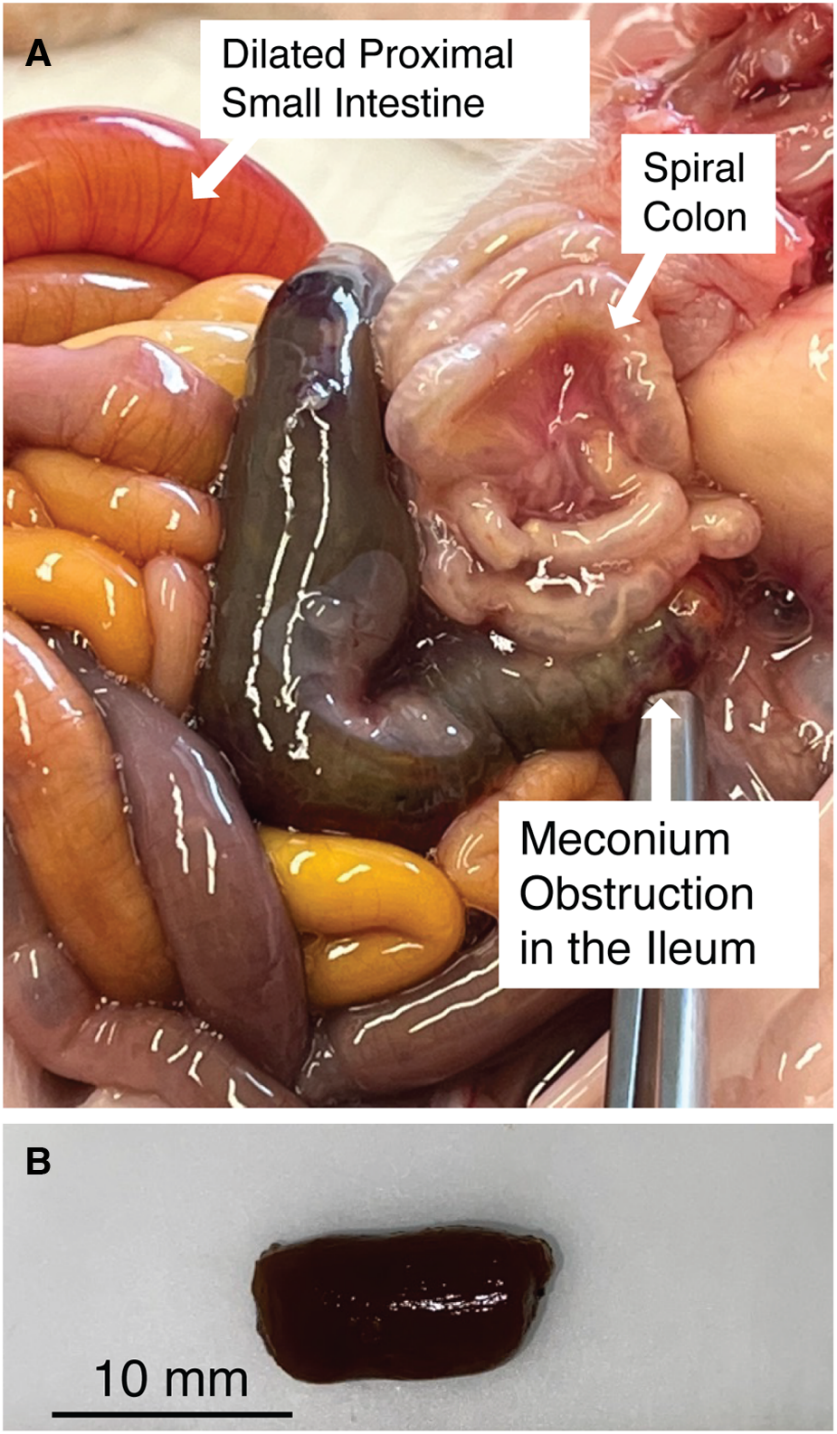
(**A**) Meconium obstruction in CF pig intestine. The proximate segment of the MI obstruction is dilated with CF meconium. The distal segment of the MI obstruction is characterized by small hypoplastic spiral colon filled by abnormal mucus The proximal small bowel is dilated by gas and liquid. (**B**) Meconium from the proximal segment of MI obstruction in CF pigs was cut into smaller pieces (5–10 mm) for digestion studies. Note that the meconium remains a congealed tubular mass even after cutting.

As previously reported, MI in CF pigs results in morphologically distinct intestinal segments (17). We observed that distal segments (i.e., farther from the oral cavity) were of small caliber (Figure 1A), while the proximal region was dilated by accumulated intestinal material and gas. The interface of these two regions has been described as the obstruction interface. This interface usually localized in the distal small intestine to proximal spiral colon in CF pigs.

### CF and WT pig meconium

We examined the meconium from newborn non-CF and CF pigs at necropsy. Because meconium from non-CF pigs was scant and typically had already passed by the time we performed necropsy, we focused on CF pig meconium. In CF pigs, we were able to identify the MI obstruction. We transected the proximal segment to expose its luminal contents. Here the meconium was congealed and retained the tubular appearance of the intestinal lumen (Figure 1B).

### Morphologic composition of meconium ileus in CF pigs

**Figure 2:**
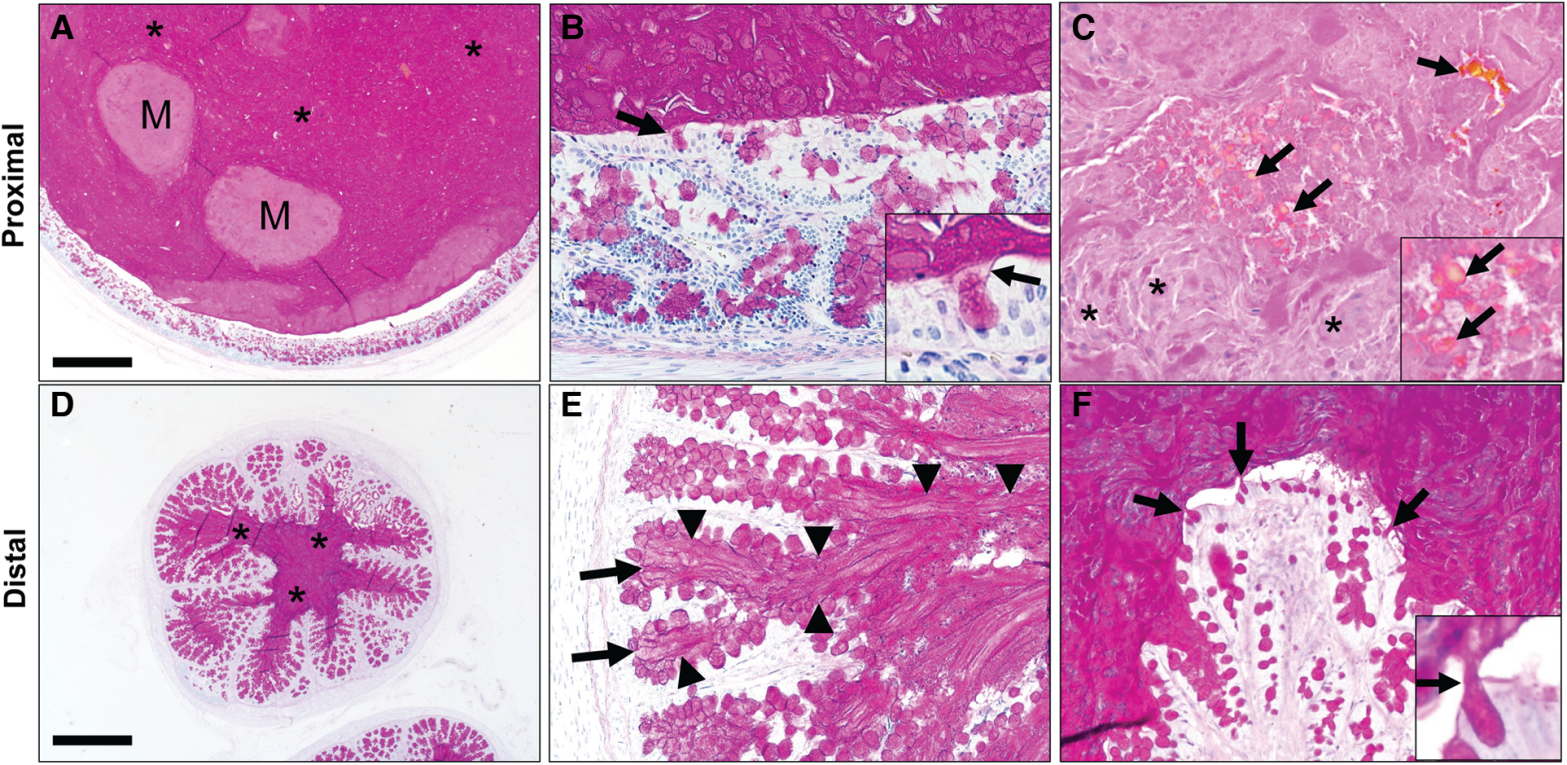
Spiral colon in newborn cystic fibrosis (CF) pigs with proximal (**A–C**) and distal (**D–F**) segments of meconium ileus obstruction. (**A**) Proximal segments of meconium ileus obstruction were composed of dPAS + mucus (*) and nascent meconium (**M**) that filled the lumen and compressed the adjacent mucosa. (**B**) dPAS + mucus was seen tethered to mucous cells (arrows, inset). (**C**) Additionally, nascent meconium contained pigmented globules consistent with bile (arrows) and sloughed cellular debris (*). (**D**) Distal segments of meconium ileus obstruction in CF pigs were composed primarily of dPAS + mucus. (**E,F**). Distal segments had dPAS + mucus that was tethered to the wall by mucous strands (arrowheads) originating from mucous cells in in the colonic crypts (arrows, **E**) and surface epithelium (arrows). Staining in all images with dPAS stain. Scale bar = 871 *µ*m (**A,D**) and 87 *µ*m (**B–F**).

We obtained paraffin-embedded blocks from archival samples (*n* = 5 pigs) to microscopically examine the intestine proximal (Figures 2A–C) and distal (Figures 2D–F) of the obstruction. The proximal segments of MI were variably dilated by accumulated intestinal contents that compressed the adjacent mucosa (Figure 2A). The luminal contents included dPAS + mucus glycoproteins as well as nascent meconium arising from the intestinal epithelium. Higher powered images revealed that glycoprotein strands of meconium were tethered to mucus cells (Figure 2B). In addition to the glycoprotein, there was dPAS- material in the lumen that was composed of sloughed cellular debris and biliary pigments (Figure 2C). The small caliber distal segments of MI were composed mostly of dPAS + mucus strands with some minor cellular debris. Distal intestinal segments had dPAS + mucus glycoproteins (Figure 2D) that arose from intestinal crypts and goblet cells (Figures 2E,F). Both proximal and distal segments of MI had dPAS + mucus exhibiting adherence to the wall, but the presence of bile pigments in the proximal segment made this region more useful in for assays evaluating the dissolution of meconium.

### Incubating meconium in aqueous solutions releases colored pigments

**Figure 3:**
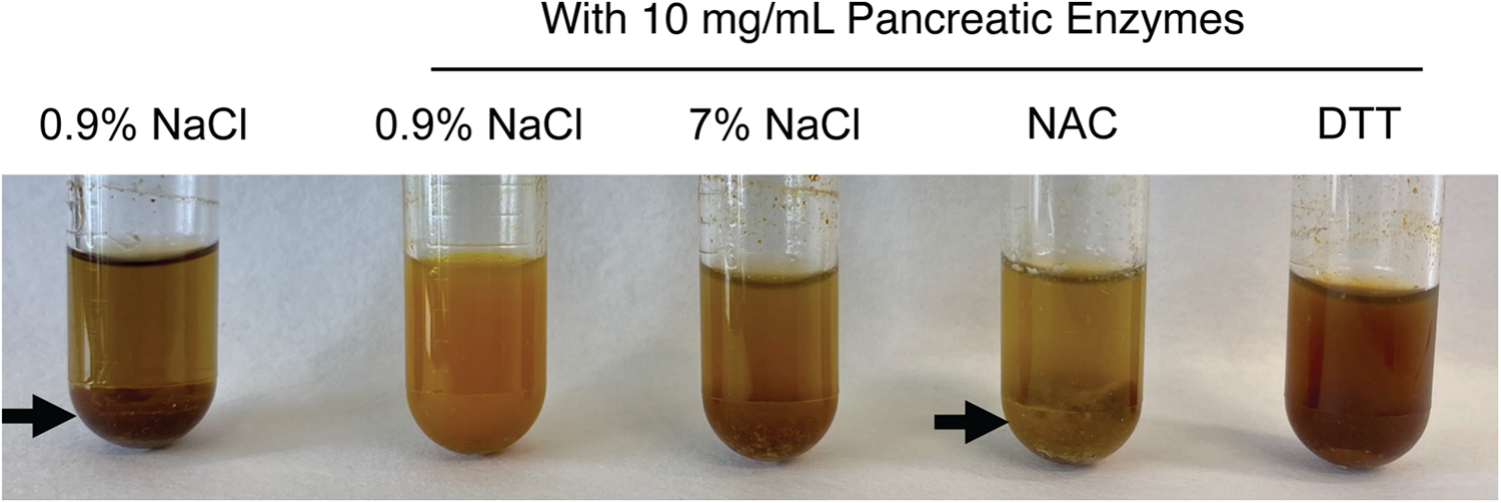
Representative photographs of meconium digestion after 16 h in different test solutions, including 0.9% NaCl without pancreatic enzymes and four test solutions containing 10 mg/ml of pancreatic enzymes. Large undigested pieces of meconium are visible in the test tubes containing 0.9% NaCl without enzymes and in the tube containing 1M NAC with pancreatic enzymes (arrows). Less residual solid meconium is visible in the tube containing pancreatic enzymes in 0.9% NaCl and in the tube containing pancreatic enzymes with 1M DTT. The contrasting results between the neutral reducing agent DTT and the acidic NAC suggests that the acidity of NAC may inhibit digestion by pancreatic enzymes.

We tested whether treatment solutions including saline, hypertonic saline, or sulfhydryl reducing agents like DTT and NAC would degrade congealed meconium originating from CF pigs. We took pieces of meconium from proximal segments of the MI obstruction, cut them into smaller fragments, and submerged them in treatment solutions. Because increased temperature or mechanical forces such as peristalsis could expedite meconium digestion, we agitated these solutions in an orbital shaker set to 225 RPM at 37 °C for 16 h. As the meconium digested, the release of biliary pigments darkened the supernatant (Figure 3). We observed that adding pancreatic enzymes increased the release of meconium pigments. Unexpectedly, we found that including 1 M NAC in solution blunted the release of meconium pigments regardless of pancreatic enzyme condition. With NAC, we found large undigested pieces of meconium remained at the bottom of the test tube.

### Meconium pigment release was greatest in samples treated with pancreatic enzymes and normal saline

**Figure 4:**
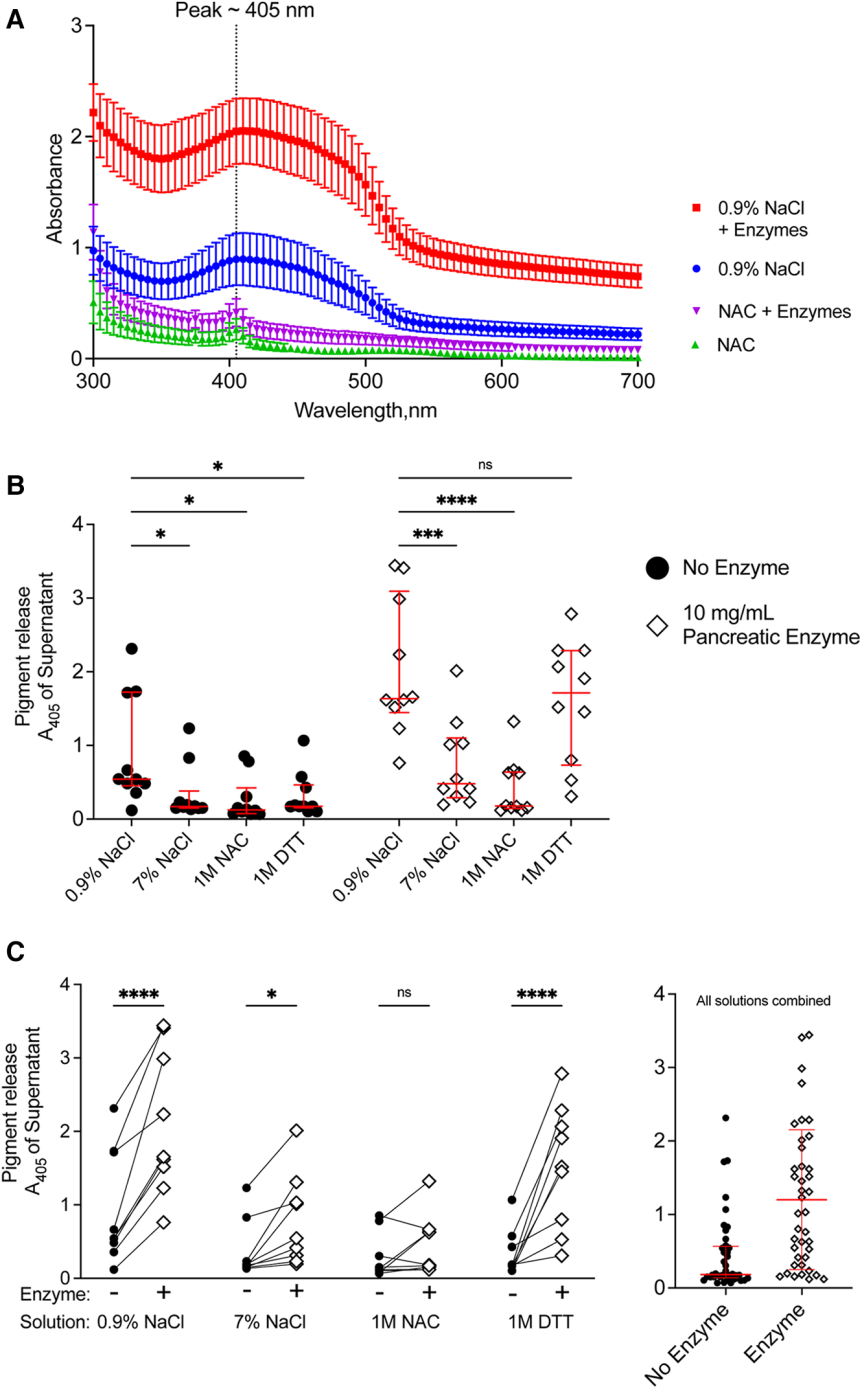
Release of pigments following digestion of CF meconium is greatest in samples treated with normal saline and pancreatic enzymes. (**A**) Absorbance spectrum of meconium pigments in supernatant following digestion in different test solutions. In each condition, the peak absorbance was near 405 nm. Pigment release was greatest when the meconium was submerged in normal saline with pancreatic enzymes. Symbols represent mean and standard error of the mean for *N* = 10 replicates. (**B**) Data show meconium pigment release, measured as A_405_, after treatment with different test solutions indicated on the x-axis. Samples with no added enzyme are grouped at left and pancreatic enzyme treated samples are grouped at right. Lines indicate median and interquartile range for each group. In the absence of pancreatic enzymes, samples treated with 0.9% NaCl had the greatest release of meconium pigment. A 2-way repeated measures ANOVA found significant effects of solution type *(p* < 0.0001), pancreatic enzyme (*p* < 0.0001) and the interaction between solution and pancreatic enzyme (*p* < 0.0001). Asterisks indicate post-hoc comparisons between groups using Holm-Sidak's multiple comparisons test. (**C**) The data are grouped by solution to show the effect of pancreatic enzyme treatment for each solution type. Each symbol indicates a different animal, lines connect samples taken from the same animal. The graph at right shows pigment release in any of the test solutions depicted as a function of pancreatic enzymes. Lines indicate median and interquartile range. Meconium pigment release increased with pancreatic enzymes in all treatment solutions except 1 M NAC. Asterisks indicate post-hoc comparisons between groups are shown using Holm-Sidak's multiple comparisons test, comparing each treatment solution vs. 0.9% NaCl. *p < 0.05, **p < 0.01, ***p < 0.001, ****p < 0.0001.

We quantified the release of meconium pigments into the supernatant by spectrophotometry. Because different solutions could alter the absorbance spectrum of the meconium pigments, we determined the absorbance of the supernatant between 300 and 700 nm. For each of the treatment solutions, there was a wide peak in absorbance near 405 nm (Figure 4A) when compared to their corresponding blank solutions. The absorbance spectrum indicates higher absorption of blue light and is consistent with the green/brown color of meconium.

As all samples had peak absorbance at 405 nm, we used this wavelength to compare the digestion by different solutions (Figure 4B). In the absence of pancreatic enzymes, treatment with normal saline resulted in the highest release of meconium pigments. There were no significant differences between any of the other solutions. This suggested that reducing agents and hypertonic saline were worse than the normal saline control in degrading meconium.

When pancreatic enzymes were added to the treatment solutions, we observed an increased absorbance of the supernatant in most of the solutions, consistent with increased meconium digestion (Figure 4C). The pancreatic enzyme effect was greatest with normal saline, followed by DTT. We observed that 1 M unbuffered NAC minimally digested meconium despite co-incubating the samples with pancreatic enzymes. The attenuation of the pancreatic enzyme digestion with NAC was not observed with DTT, suggesting that the effects of NAC on digesting meconium were unrelated to its property as a reducing agent. To test whether pH explained the inhibitory effect, we titrated NAC-containing solutions to pH 7.00 using sodium hydroxide and compared data to non-titrated NAC-containing solutions. NAC solutions with pH 7.00 digested meconium, suggesting that NAC free acid solutions inhibited pancreatic enzymes because of their acidity, Supplementary Figure S1. However, as we observed with NaCl, high solute concentrations of NAC also had poorer meconium digestion.

### Pancreatic enzymes digested meconium into small pieces with low residual weight

To relieve the obstruction caused by CF meconium, it may be necessary to break the meconium plug into smaller pieces that can pass through a narrowed intestinal lumen. We tested whether digestive solutions in the presence or absence of pancreatic enzymes could break the meconium into pieces small enough to pass through a filter. After incubating meconium in test solutions and collecting the supernatant, we poured the sediment onto pre-weighed #1 Whatman filter papers and collected residual solids by vacuum filtration. We washed filters with 50 ml of deionized water to remove any excess salt, then air-dried the filter papers. Once dry, we weighed filters on an analytical balance to calculate the residual meconium weight.

**Figure 5:**
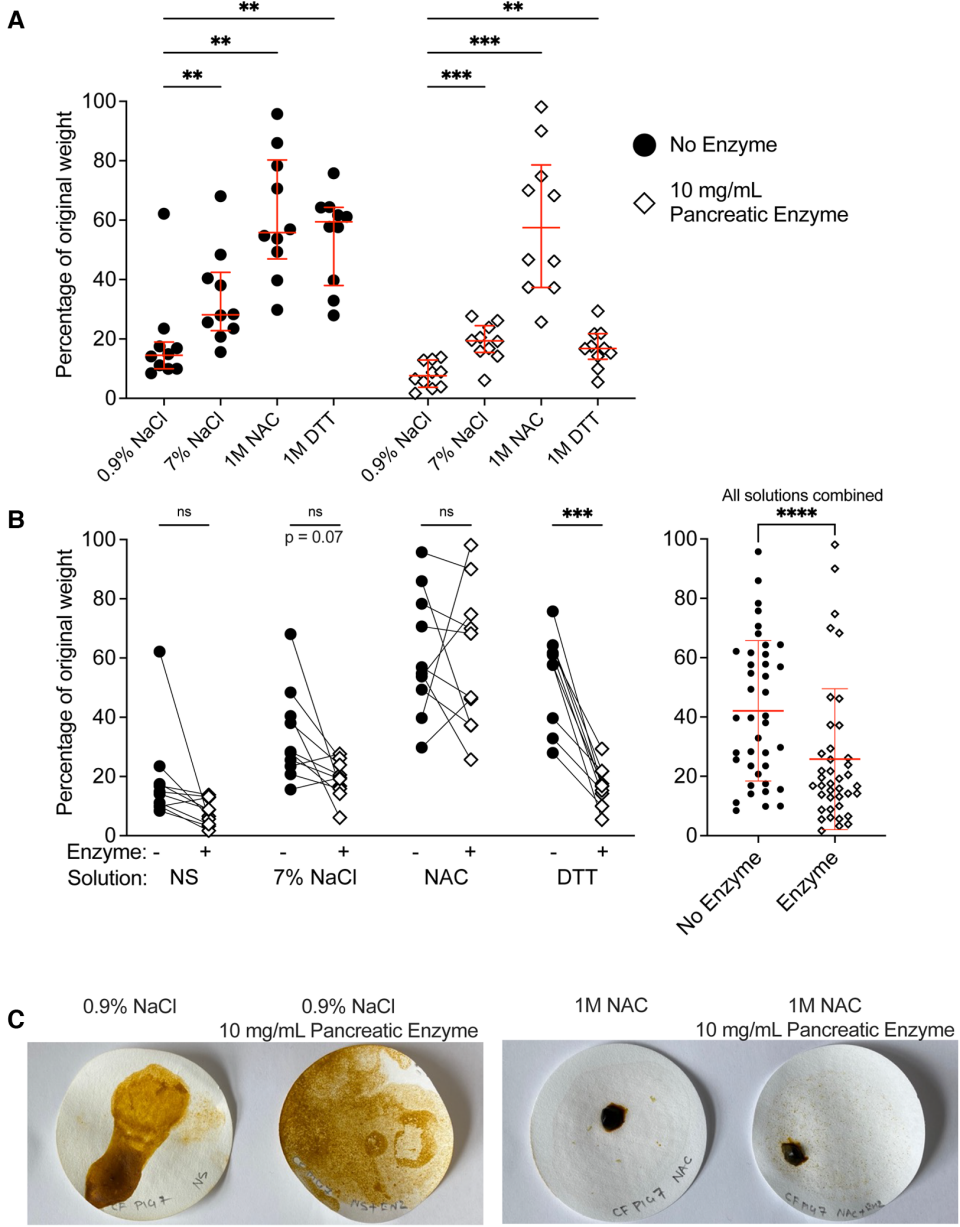
Treatment of CF meconium with pancreatic enzymes decreased residual meconium solids. (**A**) Data indicate the percentage of residual weight for meconium pieces following digestion in different test solutions and vacuum filtration. Samples with no added enzyme are grouped at left and pancreatic enzyme treated samples are grouped at right. Lines indicate median and interquartile range for each group. In the presence or absence of pancreatic enzymes, samples treated with 0.9% NaCl had the lowest residual weight. A 2-way repeated measures ANOVA found significant effects of solution type (*p* < 0.0001), pancreatic enzyme (*p* = 0.0005) and the interaction between solution and pancreatic enzyme (*p* = 0.03). *p* values for post-hoc comparisons between groups using Holm-Sidak's multiple comparisons test are given. (**B**) The data are grouped by solution to show the effect of pancreatic enzyme treatment on residual weight for each solution type. Each symbol indicates a different animal, lines connect samples taken from the same animal. The graph at right shows residual weight of meconium after treatment with any of the test solutions in the presence or absence of pancreatic enzymes. Lines indicate median and interquartile range. Asterisks indicate post-hoc comparisons between groups are shown using Holm-Sidak's multiple comparisons test, comparing each treatment solution vs. 0.9% NaCl. *p < 0.05, **p < 0.01, ***p < 0.001, ****p < 0.0001. (**C**) Representative photographs of residual meconium on the filter papers after digestion. In samples treated with NAC, there were large undigested pieces of meconium, whereas in other treatments, the residual meconium was reduced to fine pieces.

In the absence of pancreatic enzymes, we found that residual meconium weight was lowest in samples treated with 0.9% NaCl compared to the other solutions (Figure 5A). DTT and non-titrated NAC did not increase meconium degradation as measured by residual weight. This result was consistent with our observation that meconium digestion, as measured by pigment release, was best in normal saline compared with the other test solutions.

Pancreatic enzymes generally decreased the residual weight of meconium, though there were differences in the effect of pancreatic enzymes between solutions (Figure 5B). We found that pancreatic enzymes in normal saline yielded the lowest residual weight, with meconium solids decreased to approximately 10% of their original weight. Samples treated with pancreatic enzymes in the presence of 7% NaCl or 1 M DTT reduced the meconium solids to approximately 20% of their original weight, whereas pancreatic enzymes in 1M unbuffered NAC were minimally effective. These results were consistent with our studies of meconium pigment release; treatments that caused greater pigment release also decreased the weight of residual meconium.

We observed and photographed the filter papers to inspect how the meconium pieces were degraded (Figure 5C). In the specimens of meconium that were treated with normal saline and pancreatic enzymes, the meconium was degraded into fine granular particles. By contrast, the specimens treated with 1 M non-titrated NAC in the presence or absence of pancreatic enzymes had coarse pieces of residual non-filterable solid.

### Optimizing conditions for enzymatic degradation of CF meconium

Our results illustrate that pancreatic enzymes increase CF meconium digestion as measured by pigment release and weight of residual meconium solids. Because we observed differences in enzymatic digestion between 1 M NAC (an acidic reducing agent) compared to 1 M NAC titrated to pH 7.00 and 1 M DTT (a neutral reducing agent), we suspected that pH could affect enzymatic digestion. Because normal saline appeared more effective than either reducing agent or hypertonic saline, we studied the effect of pH on enzymatic digestion using an isotonic saline solution.

**Figure 6:**
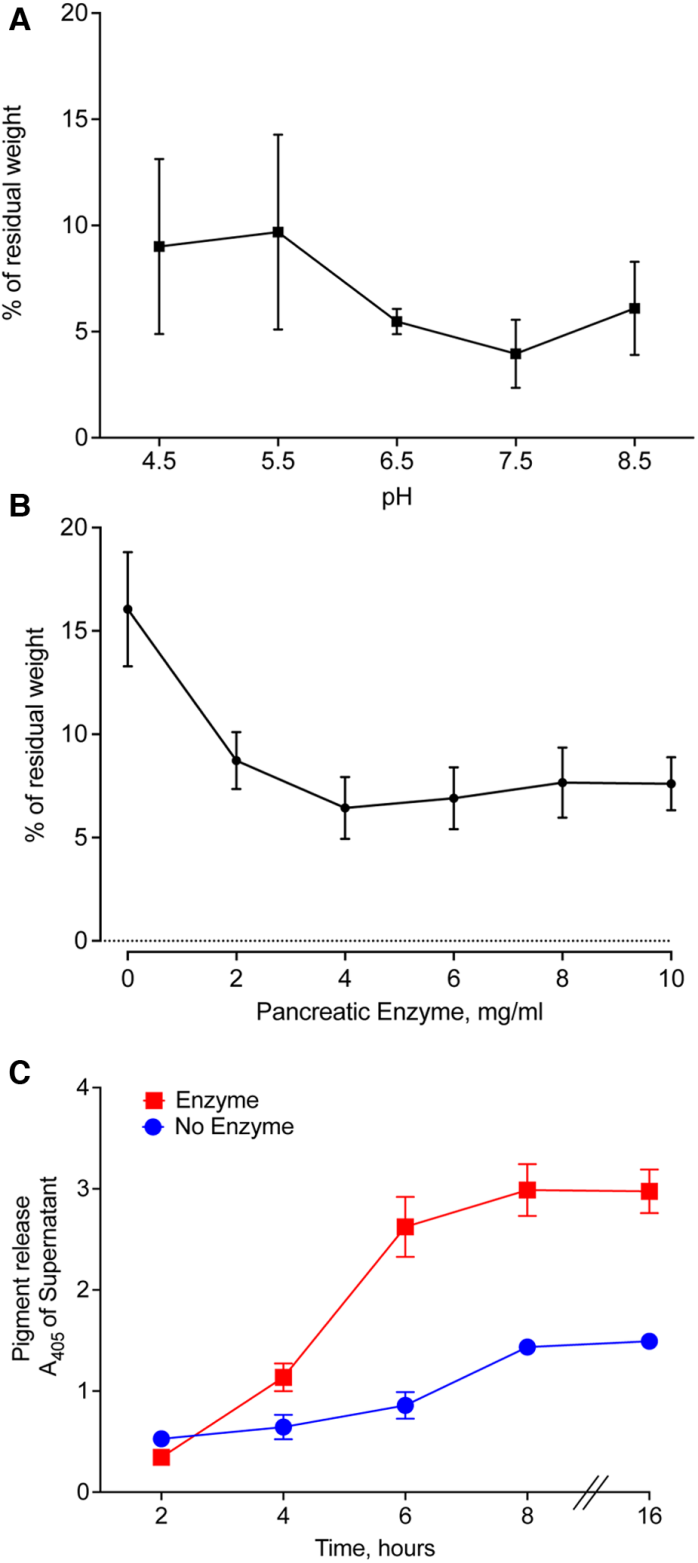
Optimal conditions for enzymatic digestion of CF meconium. (**A**) Residual meconium weight of CF meconium after pancreatic enzyme digestion varies with the pH of the test solution. The pH of the solution was established using Good's buffers with pH ranging from 4.5–8.5 and with 130 mM NaCl. Symbols represent mean and standard error of the mean for *n* = 5–6 replicates per group. There were no statistically significant differences between groups by 1-way ANOVA. The lowest observed residual meconium was at pH 7.5. (**B**) Residual weight of CF meconium following treatment with pancreatic enzymes in a dose range in 130 mM NaCl at pH 7.5. Symbols represent mean and standard error of the mean for *N* = 6 replicates per group. All doses of pancreatic enzyme were had significantly less residual meconium compared to buffer control (1-way ANOVA overall *p* = 0.002, *p* < 0.05 for all pairwise comparisons between enzyme doses and buffer control). (**C**) Time course of meconium digestion, as measured by meconium pigment release at A_405_. We observed a time-dependent increase in the release of meconium pigments, which plateaued at 6 h of digestion. Symbols represent mean and standard error of the mean for *N* = 6 replicates. A 2-way repeated measures ANOVA found significant effects of enzyme treatment (*p* = 0.0003), time (*p* < 0.0001), and interaction (*p* < 0.0001). Holm-Sidak post-hoc comparisons of enzyme-treated samples vs. control showed increased A_405_ in enzyme-treated samples at all timepoints ≥ 4 h (*p* < 0.01). Holm-Sidak post-hoc comparisons of time points in enzyme-treated samples showed no significant differences at timepoints ≥ 6 h.

We prepared pancreatic enzymes in Good's buffers with pH ranging from 4.50–8.50, added pieces of meconium, and incubated for 16 h with agitation. After filtration, we compared the weight of residual solids. The lowest residual meconium was observed in the samples treated with pancreatic enzymes at pH 7.50 (Figure 6A). A small decrease in digestion occurring at pH 8.50 suggests that pH 7.50 is near the optimal pH for digestion of meconium by pancreatic enzymes.

The amount of pancreatic enzyme could also influence the digestion of CF meconium. We tested digestion of CF meconium using pancreatic enzymes in doses ranging between 2 and 10 mg/ml and compared these to the saline buffer control at pH 7.50. We measured the residual weight after treating pieces of meconium. All doses had lower residual weight compared to the buffer control. The dose response was plateaued beyond 4 mg/ml (Figure 6B).

Autodigestion by pancreatic enzymes could limit their duration of action, and it is unclear how long meconium needs to be treated for complete digestion. Therefore, we studied pancreatic enzymes in a time course to determine how long they continue to digest meconium. We selected 4 mg/ml pancreatic enzymes in the saline buffer at pH 7.5 for our treatment condition. We elected to use pigment release for this portion of the study as we needed to repeatedly sample from the digestion reactions. We found that pancreatic enzymes caused an increase in meconium pigment release within 4 h compared to buffer control (Figure 6C). By 6 h, there were no further significant increases in meconium pigment release. This suggests that digestion slows down after approximately 6 h.

## Discussion

Currently, MI is medically managed by administering therapeutic solutions rectally, but a standard protocol for treatment does not exist (18). Preferred treatment varies between institutions depending on physician preference and experience. Thus, many different enema solutions are used around the world including normal saline, iso-osmolar contrast materials (Omnipaque, Cysto-Conray II), hyperosmolar contrast material (Gastrografin), and NAC (1, 7, 19). Regardless of the treatment chosen, at least half of MI cases do not respond to these enemas. In those cases, surgical intervention is required to relieve the obstruction.

This suggested that existing strategies for treating MI are not effective, possibly because they do not sufficiently degrade obstructive meconium. We found that NAC, a commonly used mucolytic enema agent that works by reducing disulfide bonds (20), caused minimal breakdown of the meconium when administered as a free acid. Neutral-buffered NAC degraded meconium better than NAC as a free acid but did not improve meconium breakdown vs. saline or pancreatic enzymes. The neutral reducing agent DTT, which has two thiol groups, also did not fully degrade obstructive meconium when given in the absence of pancreatic enzymes and had no advantage over normal saline.

Most patients with CF have exocrine pancreatic insufficiency and some cases of MI that develop in the absence of CF suggest a role for the pancreas in the pathophysiology of MI (23–25). Bishop and Koop described the potential role of pancreatic enzymes in MI in 1957, but since then we found no further studies examining their utility in treating MI (21). There is strong mechanistic rationale for using pancreatic enzymes; meconium contains glycoproteins, a typical substrate of pancreatic enzymes. We tested a pancreatic enzyme formulation approved by the FDA for treating exocrine pancreatic insufficiency in animals. This formulation digested meconium best at neutral pH and without high salt or reducing agent.

While the CF pig has been useful to study MI (17), the pathophysiology of MI remains incompletely understood. Possible pathophysiologic mechanisms for MI include a lack of CFTR function in the intestinal epithelial cells causing (a) abnormal pH or HCO_3_^−^ concentration in the intestinal lumen, (b) decreased hydration of the meconium making it more solid than in the non-CF intestine, (c) abnormal mucin cross-linking, (d) CFTR-dependent smooth muscle dysfunction causing intestinal dysmotility, or (e) CFTR-dependent intestinal growth anomalies leading to atresia, malrotation, or other structural abnormalities. It is possible that a combination of these factors contribute to physical obstruction of the distal small intestine (1, 3, 17, 22–27). Our data suggest that the lack of pancreatic enzyme activity could contribute to MI.

### Study advantages

Though mice lacking CFTR have been used as a model for the CF gastrointestinal tract and pancreas, they lack MI development but instead develop a distal intestinal obstruction syndrome-like phenotype postnatally without dietary modifications (28–30). An advantage of our study was that we used CF pigs, which uniformly develop MI similar to that seen in humans with 100% penetrance along with the associated organ dysfunction, making them an excellent model for our study (4, 14, 15). Additionally, we consistently collected CF pig meconium from the proximal segment of the MI obstruction to better quantify our assays and treatments using the biliary pigments that accumulated there. Using CF pigs also gave regular access to a reproducible source of meconium for testing, which would not have been possible with clinical samples or in other animal models.

### Study limitations

Our experiments were performed ex vivo and may not reflect efficacy *in vivo*. We have not demonstrated the effect of individual components of the pancreatic enzyme mixture. However, we specifically chose to use a mixed pancreatic enzyme formulation because FDA-approved enzyme treatments are available for pancreatic insufficiency. We collected samples for testing exclusively from the distended portions proximal to the obstruction. therefore, it is possible that obstructions in other segments have different digestion characteristics.

The dosing of pancreatic enzymes required to relieve intestinal obstruction *in vivo* will require further investigation. At our lowest concentration of 4 mg/ml, the mixture included approximately 100 lipase units/ml and 550 protease units/ml. The typical dosing of pancreatic enzymes for infants receiving a 120 ml feeding is 2,000–4,000 lipase units, with the high end of 33 lipase units/ml (31). Meconium may be more difficult to digest, as shown by the higher concentrations in our studies.

Finally, our experiments relied on a mechanical shaker to agitate the meconium and assure adequate mixing. It is not known whether there are adequate smooth muscle contractions in the CF intestines to physically decompose meconium. Therefore, the mechanical requirements for breaking down meconium need further exploration in future studies.

## Conclusions

Our results show that isotonic solutions are more effective than hypertonic solutions for the digestion of CF pig meconium, a finding that may inform the use of isotonic enema solutions and potentially decrease complications such as dehydration, hypovolemic shock, and even death that can be associated with hypertonic enemas. Our study also shows no significant effects of reducing agents on digestion of CF meconium. Determining whether current therapies are effective or not may help inform the management of MI in newborns with CF, sparing them from exposure to ineffective and potentially toxic treatments. Finally, our study suggests a role for exocrine pancreatic function in the development of MI.

## Data Availability

The original contributions presented in the study are included in the article/**Supplementary Material**, further inquiries can be directed to the corresponding authors.
