# Supplementary material for: Pancreatic enzymes digest obstructive meconium from cystic fibrosis pig intestines
Source: Front Pediatr. 2024 Apr 11;12:1387171. doi: 10.3389/fped.2024.1387171 (PMC11043547; doi:10.3389/fped.2024.1387171)
Supplement: Supplementary file 1 [file Datasheet1.pdf]

Data Supplement for
Pancreatic enzymes digest obstructive meconium
from cystic fibrosis pig intestines

by

Gopinathan Gangadharan Nambiar[1,2], Sussette Gonzalez[1], Christian Zirbes[1], Linda Powers[3], David K. Meyerholz[4], Ian Thornell[3], David A. Stoltz[3], Anthony J. Fischer[1]

Supplemental Table 1. Individual pigs used in this study.

Pig Number	Sex	Genotype	Experiment/Figure
552-A13366	M	*CFTR* $^{-/-}$	Screening solutions for meconium pigment release, Figure 3. Screening solutions for residual meconium solids, Figure 4.
552-A13367	M	*CFTR* $^{-/-}$	Figure 3, Figure 4
552-A13370	M	*CFTR* $^{-/-}$	Figure 3, Figure 4
552-A13373	F	*CFTR* $^{-/-}$	Figure 3, Figure 4
553-A13385	M	*CFTR* $^{-/-}$	Figure 3, Figure 4
553-A13391	F	*CFTR* $^{-/-}$	Figure 3, Figure 4
556-A13625	M	*CFTR* $^{-/-}$	Figure 3, Figure 4
556-A13631	F	*CFTR* $^{-/-}$	Figure 3, Figure 4
567-A13643	F	*CFTR* $^{-/-}$	Figure 3, Figure 4
567-A13647	F	*CFTR* $^{-/-}$	Figure 3, Figure 4
654-B1680	M	*CFTR* $^{-/-}$	pH dependence of meconium digestion, Figure 5A
580-A13797	M	*CFTR* $^{\Delta F508/\Delta F508}$	Figure 5A
583-A13816	F	*CFTR* $^{-/-}$	Figure 5A

Pig Number	Sex	Genotype	Experiment/Figure
585-A13853	F	*CFTR* $^{-/-}$	Figure 5A
595-A13960	M	*CFTR* $^{-/-}$	Figure 5A
595-A13868	F	*CFTR* $^{-/-}$	Figure 5A
575-A13733	M	*CFTR* $^{-/-}$	Enzyme dose response for meconium digestion, Figure 5B
575-A13735	M	*CFTR* $^{-/-}$	Figure 5B
575-A13737	F	*CFTR* $^{-/-}$	Figure 5B
576-A13747	M	*CFTR* $^{-/-}$	Figure 5B
577a-A13768	F	*CFTR*$^{\Delta F508/\Delta F508}$	Figure 5B
578-A13775	F	*CFTR* $^{-/-}$	Figure 5B
638-B1440	M	*CFTR* $^{-/-}$	Kinetics of meconium digestion, Figure 5C
638-B1443	F	*CFTR* $^{-/-}$	Figure 5C
653-B1664	M	*CFTR* $^{-/-}$	Figure 5C
653-B1665	M	*CFTR* $^{-/-}$	Figure 5C
653-B1666	M	*CFTR* $^{-/-}$	Figure 5C
654-B1680	M	*CFTR* $^{-/-}$	Figure 5C
743-B4522	F	*CFTR* $^{-/-}$	Determining whether buffering NAC increases meconium digestion compared to NAC as free acid, Supplemental Figure 1
743-B4524	F	*CFTR* $^{-/-}$	Supplemental Figure 1
747-B4538	F	*CFTR* $^{-/-}$	Supplemental Figure 1
747-B4539	F	*CFTR* $^{-/-}$	Supplemental Figure 1

Supplemental Figure 1.

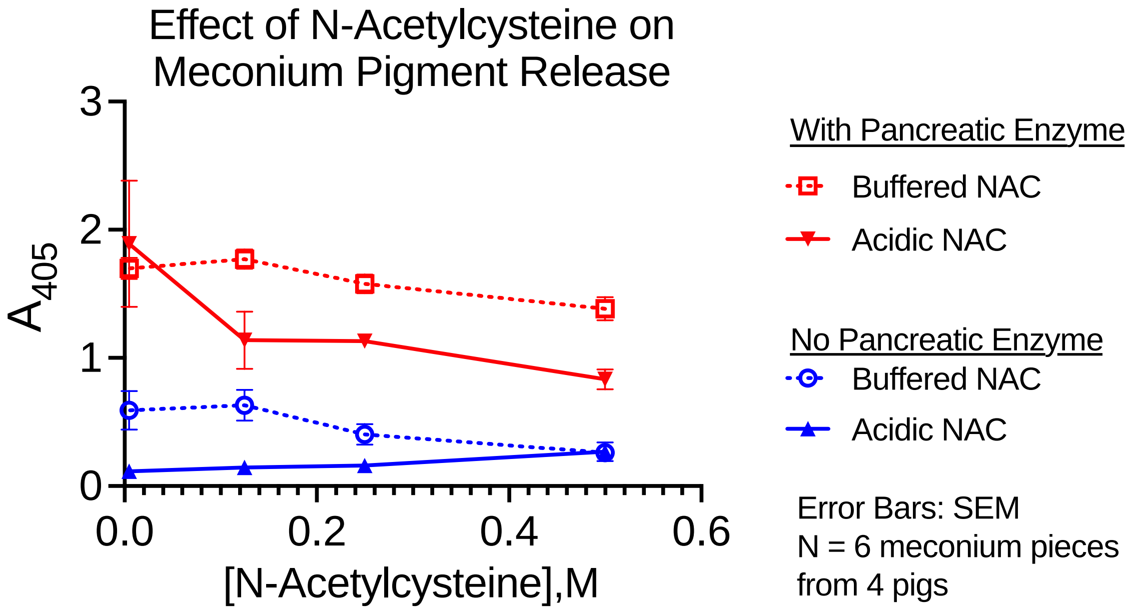

The A405 of supernatant following 16-hour digestion of CF meconium is displayed versus N-Acetylcysteine (NAC) concentration. Symbols represent mean from *n* = 6 experiments with meconium pieces derived from 4 CF pigs; Error bars are standard error of the mean. Data are grouped by treatment condition. Red indicates experiments with 10 mg/mL of pancreatic enzymes, blue without pancreatic enzymes. Open symbols indicate NAC was titrated to pH 7.00; closed symbols represent NAC was used as a free acid. We analyzed the data using a repeated measures 3-way ANOVA with enzyme, NAC concentration, and buffering as factors. Pigment release from CF pig meconium was increased by pancreatic enzymes ($P < 0.0001$). Buffering N-Acetylcysteine (NAC) at a pH of 7.50 increased the release of meconium pigments compared to NAC as a free acid ($P = 0.03$), but higher NAC concentrations decreased the release of meconium pigments ($P = 0.003$).
